# Anti‐retroviral therapy scale‐up and its impact on sex‐stratified tuberculosis notification trends in Uganda

**DOI:** 10.1002/jia2.25394

**Published:** 2019-09-16

**Authors:** Stella Zawedde‐Muyanja, Yukari C Manabe, Joseph Musaazi, Frank R Mugabe, Jennifer M Ross, Sabine Hermans

**Affiliations:** ^1^ The Infectious Diseases Institute College of Health Sciences Makerere University Kampala Uganda; ^2^ Division of Infectious Diseases Department of Medicine Johns Hopkins University School of Medicine Baltimore MD USA; ^3^ Ministry of Health National Tuberculosis and Leprosy Program Kampala Uganda; ^4^ Departments of Global Health and Medicine University of Washington Seattle WA USA; ^5^ Department of Global Health Amsterdam Institute for Global Health and Development Amsterdam UMC University of Amsterdam Amsterdam The Netherlands

**Keywords:** antiretroviral therapy, tuberculosis, sex, policy, notification trends, Uganda

## Abstract

**Introduction:**

In order to end the tuberculosis (TB) epidemic by 2035, countries must achieve a 10% annual decline in tuberculosis incidence rates by 2025. Provision of antiretroviral therapy (ART) has been associated with population level decreases in TB notification rates. We aimed to assess whether the progressive scale‐up of ART provision over the past nine years has had an effect on population level trends of TB notification in Uganda stratified by sex and HIV status.

**Methods:**

The study area consisted of Kampala and eight surrounding districts. Annual TB notifications and mid‐year populations were used to calculate notification rates per 100,000 population from the study area. Numbers alive and retained on ART were used to calculate ART coverage, overall and by sex. TB notification rates (TBNRs) overall and stratified by sex and HIV status were calculated for the period 2009 to 2017. Trends in TBNRs before and after rollout of universal ART for pregnant women in 2013 were examined using Poisson regression models. To gain insight into the trends in CD4+ T‐cell counts at ART initiation over the study period, we performed a sub analysis of patient level data from the Infectious Diseases Institute clinic.

**Results:**

From 2009 to 2017, ART coverage increased by 27.6% among men and by 35.4% among women. TBNRs declined during the same period. Overall, the average annual percentage decline in TBNRs was −3.5% (95%CI −3.7% to −3.3%), (−2.3% (95%CI −2.6% to −1.9%) in men and −5.4% (95%CI −5.7% to −5.0%) in women). ART coverage increased after 2013 but this was not associated with an accelerated decline in overall TBNRs among HIV‐positive persons −3.6% before 2013 and −5.2% after 2013; p = 0.33. The proportion of patients initiating ART with CD4+ T‐cell count ≤ 200 cells/mL did not decrease significantly after 2013 (42.2% to 32.2%*, p = *0.05).

**Conclusions:**

Although ART scale‐up was temporally associated with a decline in TB notification rates, the achieved rates of decline are below those required to achieve the End TB Targets. Additional investments in tuberculosis control should include efforts to promote earlier care seeking and ART initiation among HIV‐positive persons.

## 1 Introduction

One of the Sustainable Development Goals (SDGs), incorporated in the End TB Strategy, is to end the global tuberculosis (TB) epidemic by 2030 1, 2. In order to realize this, the World Health Organization (WHO) estimates that the annual decline in global incidence rates must accelerate from 2% in 2015 to 10% by 2025 2.

In sub‐Saharan Africa (SSA), high rates of HIV prevalence present a substantial challenge to achieving the SDGs because HIV infection increases the risk of reactivation of latent TB 3, progression to active disease following TB infection 3, 4, and recurrence after treatment 5, 6 Eastern and Southern Africa accounts for 53% of the global burden of persons living with HIV (PLHIV) and 45% of global new HIV infections annually 7. In Uganda, it is estimated that 50,000 people became newly infected with HIV in 2017 7. The subsequent high HIV burden results in an increased TB burden and subsequently increased TB transmission at the community level.

The provision of antiretroviral therapy (ART) is one of the four key interventions recommended by the WHO to reduce the burden of TB among HIV‐positive persons 8. ART has powerful protective effects against incident TB and mortality from TB among persons living with HIV (PLHIV). Among treatment cohorts of PLHIV, ART reduces TB incidence by 67% and mortality by 65% to 90% 9, 10. At the community level, the rapid scale‐up of ART in Brazil and South Africa was associated with significant decreases in TB notifications 11, 12. Mathematical modelling studies investigating the long‐term impact of ART on the population level incidence of TB indicate that ART has the potential to reduce the incidence of HIV‐associated TB by more than 90% with greater decreases anticipated if ART is initiated soon after HIV infection 13, 14 ^.^

In Uganda, HIV infection is one of the major drivers of TB disease. The country ranks 10th among the 30 high TB‐HIV burden countries and in 2017, 44% of TB patients notified to the National TB and Leprosy Programme (NTLP) were co‐infected with HIV 15. Large‐scale provision of ART in public health facilities across the country started in 2008 and has substantially increased since. We set out to examine whether the progressive scale‐up of ART provision over the past nine years has had an effect on population level trends of TB notification in Uganda and whether this effect differs by sex and/or HIV status.

## 2 Methods

### 2.1 Study setting

The study area consisted of Kampala and eight surrounding districts (Buikwe, Butambala, Buvuma, Gomba, Luweero, Mpigi, Mukono and Wakiso). The districts surrounding Kampala were included to provide a complete representation of the Kampala catchment area that caters for the frequent movement of people from neighbouring districts into the capital city for healthcare services. Data from the study area for the period 2009 to 2017 were analysed for this study. The combined estimated mid‐year population of the study area in 2017 was 6.1 million 16. During the same year, the overall HIV prevalence was estimated at 9.0% and was higher among women (10.7%) than men (7.2%) 17 The number of PLHIV was estimated at 550,000.

ART eligibility varied during the study period: from 2009 to 2011, ART was offered to persons with CD4+ T‐cell <250/mm 18. In 2012, ART eligibility was increased to CD4+ T‐cell count <350 cells/mm 19. From 2013 onwards, following the adoption of the universal drive to eliminate mother‐to‐child transmission (eMTCT) of HIV, all pregnant mothers were offered ART regardless of CD4+ T‐cell count while ART eligibility for the rest of the population was maintained at CD4+ T‐cell count <350 cells/mL 20. In 2014, ART eligibility was increased to CD4+ T‐cell counts <500 cells/mL for the general population; in addition, ART was offered to all HIV‐positive persons belonging to specific populations, for example, patients diagnosed with TB and patients co‐infected with Hepatitis B 21. Starting from 2016, ART is offered to all HIV‐positive persons regardless of CD4 + T‐cell count in a policy known as “test and treat” 22. TB diagnosis is carried out using light or fluorescence microscopy (FM) where available. In 2011, the NTLP decreased the number of sputum examinations required for TB diagnosis from three to two 19. From 2013, Xpert MTB/RIF testing was introduced into the study area and by 2016 was the recommended first test for all HIV‐positive patients with signs and symptoms of TB 23.

### 2.2 Study design and population

This was a retrospective analysis of data on TB notifications and ART coverage (proportion of PLHIV alive and retained on ART each year) of the study area as reported by the NTLP and the ACP of the Uganda Ministry of Health.

For the study period considered, data on CD4+ T‐cell counts at ART initiation were not centrally available at the ACP. To gain insight into the trend of CD4+ T‐cell counts at ART initiation, we analysed patient level data from a subset of the study population receiving care from the IDI clinic. The IDI clinic is located within Mulago National Referral Hospital in Kampala and provides free HIV treatment to over 8000 patients.

### 2.3 Data sources and collection procedures

The overall and sex‐specific population estimates for the study area were obtained from the national population censuses carried out in 2002 and 2014 16, 24.The annual mid‐year populations for 2003 to 2013 were estimated by adjusting the population for each year after 2002 using the annual inter‐census population growth between the two national population censuses. The annual mid‐year populations for 2015 to 2017 were calculated using the estimated annual population growth provided by the 2014 national population census.

The overall and sex‐specific HIV prevalence estimates were obtained from the national AIDS indicator surveys carried out in 2005 and 2011 25, 26. For 2015, weighted prevalence estimates (provided by the CDC) were used. For 2007 to 2010, the annual HIV prevalence for the study area was estimated by adjusting the prevalence for each year after 2006 using the annual inter‐survey increase in HIV prevalence between 2006 and 2011. For 2012 to 2014, the annual HIV prevalence for the study area was estimated by adjusting the prevalence for each year after 2011 using the annual increase in HIV prevalence estimates between 2011 AIDS Indicator Survey and the 2015 weighted district prevalence. Annual increases for 2012 to 2014 were applied to 2016 and 2017.

Numbers of TB patients notified in the study area, between 2009 and 2017 were abstracted from the quarterly TB notification reports submitted to the NTLP. The quarterly reports provide data in aggregate on the number of TB cases notified, their age, sex, type of TB, history of TB treatment and HIV co‐infection. Double counting is avoided by only reporting on patients diagnosed at health facilities and excluding those transferred in from a different health facility or district during TB treatment. The number of people alive and retained on ART in the study area was abstracted from the annual reports published by the AIDS Control Programme (ACP).

Data on CD4+ T‐cell counts at ART initiation were obtained from the IDI clinic database which is a provider based electronic medical records system where data from each patient visit is stored 27. The system correlates clinic data with laboratory and pharmacy records.

The study utilized de‐identified aggregated data collected as part of routine programme monitoring and evaluation and was granted a waiver from obtaining informed consent by the School of Medicine Research and Ethics Committee of the Makerere University College of Health Sciences and by the Uganda National Council for Science and Technology. The IDI clinic holds a consent waiver to analyse routinely clinic collected clinic data after removing patient identifiers. This waiver is renewed annually.

### 2.4 Data management and analysis

**Figure 1:**
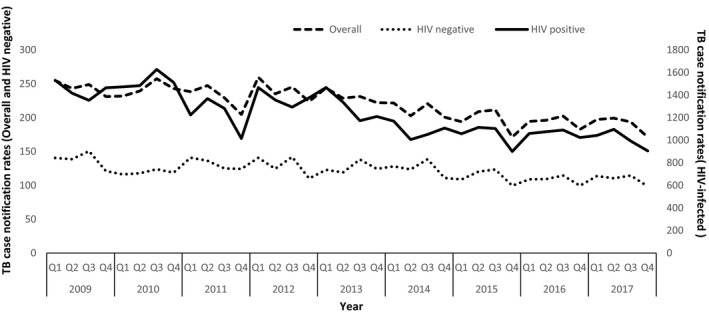
TB case notification rates per 100,000 population; overall and stratified by HIV status for the study population (2009 to 2017).

**Figure 2:**
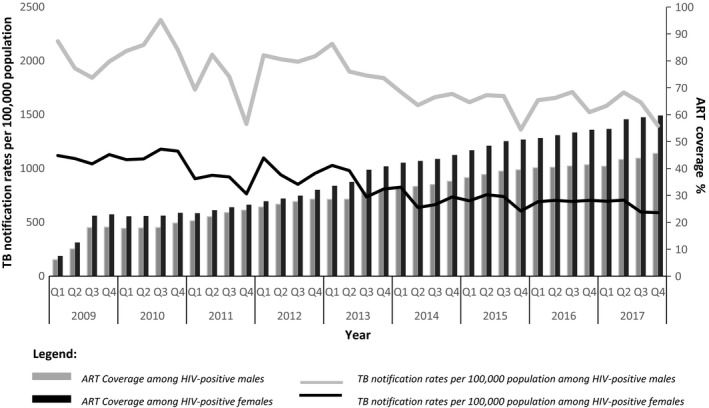
TB notification rates and ART coverage among HIV‐positive persons stratified by sex for the period 2009 to 2017.

Data were extracted and entered into a Microsoft Excel worksheet. Data analysis was done using STATA 14.2 TX, USA. Per year, we calculated the number of PLHIV in the study area by applying the HIV prevalence to the mid‐year population of the districts. We calculated the proportion of PLHIV by dividing the number of PLHIV in the study area over the total population of this area. We calculated ART coverage as the number of PLHIV who were alive and retained on ART at the end of each year divided by the estimated number of PLHIV in that year. We then calculated the overall TB NRs for each year by dividing the total number of TB cases notified by the respective estimated mid‐year population to obtain notifications per 100,000 population with their 95% confidence intervals (95% CI). The NRs stratified by sex and HIV status were also obtained using the same method. We calculated male: female TBNR overall and stratified by HIV status. We then plotted TB NRs, overall and stratified by sex and HIV status, against time in quarters for the study period (Figures 1 and 2). We also calculated the percentage changes in notification rates between the years.

We modelled linear trends in notification rates over time using a Poisson generalized linear model adjusted for heteroscedasticity and autocorrelation. To examine the impact of the adoption of universal eMTCT among HIV‐positive persons, we used piecewise regression with linear splines inserted at quarter one 2013 to investigate the effect on TB notification rates of the differential increase in ART coverage between the sexes.

From the IDI clinic records, we extracted data from all patients who presented for HIV care and initiated ART from January 2009 to December 2017. For our analysis, we considered CD4+ T‐cell counts recorded ≤ 3 months before ART initiation as the baseline CD4+ T‐cell count. We calculated the median baseline CD4+ T‐cell and interquartile range (IQR) for each year overall and stratified by sex. We then compared the trend in baseline CD4+ T‐cell count before and after 2013 for both sexes using Cuzick's test for trend 28. We calculated the proportion of patients initiating ART with advanced HIV disease (CD4+ T‐cell counts ≤ 200 cells/mL) 29 each year overall and stratified by sex. We then compared the trend in the proportion of patients presenting with advanced HIV disease before and after 2013 for both sexes using Royston's test for trend 30.

## 3 Results

From 2009 to 2017, the estimated mid‐year population of Kampala and its eight surrounding districts increased from 4842,713 to 6145,582 (Table 1). During the same period, the proportion of PLHIV increased from 8.2% (95% CI 8.2 to 8.2) to 9.0% (95% CI 8.9 to 9.0). ART coverage increased from 20.0% (95% CI 19.8 to 20.1) in 2009 to 51.5% (95% CI 51.4 to 51.6) by the end of 2017. The proportions of PLHIV who were enrolled on ART were higher for women than for men throughout the study period. The proportion of men enrolled on ART increased from 18% in 2009 to 46% in 2017 while the proportion of women enrolled on ART increased from 22% in 2009 to 57% in 2017 (Table 1).

**Table 1:** Estimated mid‐year population, number of persons living with HIV and art coverage for the study population

Year	Midyear population		No. (%) PLHIV		No. (%) on ART	
	M	F	M	F	M	F
2009	2,367,321	2,475,392	154,071 (6.5%)	244,317 (9.9%)	28,433 (18%)	52,533 (22%)
2010	2,447,317	2,558,254	165,715 (6.8%)	275,226 (10.8%)	33,117 (20%)	59,604 (22%)
2011	2,527,313	2,641,117	179,648 (7.1%)	286,918 (10.9%)	44,566 (25%)	73,751 (26%)
2012	2,607,309	2,723,979	184,964 (7.1%)	294,277 (10.8%)	53,514 (29%)	91,431 (31%)
2013	2,684,589	2,803,884	191,966 (7.2%)	303,073 (10.8%)	61,833 (32%)	117,756 (39%)
2014	2,767,302	2,889,704	197,329 (7.2%)	311,969 (10.8%)	70,513 (36%)	135,218 (43%)
2015	2,847,298	2,972,567	204,266 (7.2%)	320,077 (10.8%)	81,572 (40%)	156,899 (49%)
2016	2,927,294	3,055,429	210,240 (7.2%)	329,199 (10.8%)	87,917 (42%)	168,751 (51%)
2017	2,367,321	2,475,392	216,215 (7.2%)	338,320 (10.8%)	99,571 (46%)	192,555 (57%)

ART, antiretroviral therapy; PLHIV, persons living with HIV.

Stratified by sex, the ratio of TB notification rates among men compared to women was 1.6:1. Stratified by HIV status, the ratio of notification rates among men compared to women was 2.2:1 among PLHIV and 1.8:1 among HIV‐negative persons (Table S1).

Overall, TB notification rates decreased over the study period with an average annual change of −3.5% (95%CI, −3.7% to −3.3%) (Figure 1). Stratified by sex and HIV status the average annual percentage decline among HIV‐negative persons was −2.6% (−1.8% among men and −4.1% among women, Table 2. Among PLHIV, the average annual percentage decline was −5.0% (−3.7% among men and −7.0% among women, Table 2).

**Table 2:** Annual TB notification rates per 100,000 population and per cent change in rates overall an stratified by HIV status

Year	Overall population				HIV‐negative persons				HIV‐positive persons			
	Male		Female		Male		Female		Male		Female	
	NR (95%CI)	% rate change	NR (95%CI)	% rate change	NR (95%CI)	% rate change	NR (95%CI)	% rate change	NR (95%CI)	% rate change	NR (95%CI)	% rate change
2009	288 (281 to 295)		203 (196 to 208)		170 (164 to 175)		105 (104 to 108)		1986 (1916 to 2057)		1096 (1055 to 1188)	
2010	288 (281 to 294)	−0.1	200 (194 to 205)	−1.4	150 (145 to 155)	−11.5	88 (83 to 92)	−16.3	2180 (2109 to 2252)	9.8	1129 (1089 to 1169)	3.0
2011	279 (273 to 285)	−3.2	183 (177 to 187)	−8.5	165 (160 to 170)	+9.8	97 (93 to 101)	+11.5	1763 (1702 to 1825)	−19.1	881 (847 to 916)	to 21.9
2012	297 (290 to 303)	+6.6	187 (181 to 191)	+2.2	165 (160 to 170)	−0.0	93 (89 to 97)	−4.6	2025 (1960 to 2091)	14.9	961 (926 to 997)	9.1
2013	282 (275 to 287)	−5.2	183 (178 to 188)	−1.9	154 (149 to 158)	−6.9	98 (94 to 102)	+5.4	1939 (1877 to 2002)	−4.2	888 (854 to 921)	−7.7
2014	267 (261 to 273)	−5.1	158 (153 to 162	−14.0	160 (155 to 164)	+3.9	90 (86 to 94)	−7.9	1664 (1608 to 1721)	−14.2	715 (686 to 745)	−19.4
2015	253 (247 to 259)	−6.3	140 (137 to 146)	−10.7	149 (144 to 154)	−6.9	75 (71 to 78)	−17.9	1562 (1509 to 1617)	−6.1	694 (666 to 724)	−3.0
2016	249 (243 to 254)	−0.5	141 (136 to 145)	+0.0	142 (138 to 147)	−4.5	74 (73 to 77)	−0.7	1631 (1576 to 1686)	4.4	698 (669 to 727)	0.6
2017	247 (241 to 253)	−0.9	136 (132 to 139)	−3.6	144 (140 to 149)	+1.4	74 (71 to 77)	+0.7	1574 (1522 to 1627)	−3.5	646 (619 to 674)	−7.4
Av. annual % change		−2.28		−5.38		−1.79		−4.06		−3.71		−7.02

95% CI, confidence Interval; NR, notification rate per 100,000 population.

**Table 3:** Average annual percentage changes (and 95% confidence intervals) in TB notification rates among PLHIV pre‐ and post‐2013

	Before 2013 annual percentage change (95% CI)	After 2013 annual percentage change (95% CI)	*p*‐value
Overall	−3.6% (−5.6% to −1.6%)	−5.2% (−7.5% to −2.8%)	0.33
Males	−1.5% (−3.5% to +0.6%)	−4.4% (−6.7% to −2.0%)	0.09
Females	−6.2% (−8.1% to −4.3%)	−6.6% (−8.9% to −4.2%)	0.84

CI, confidence interval; P‐values were generated using Wald tests comparing rates before and after 2013.

**Figure 3:**
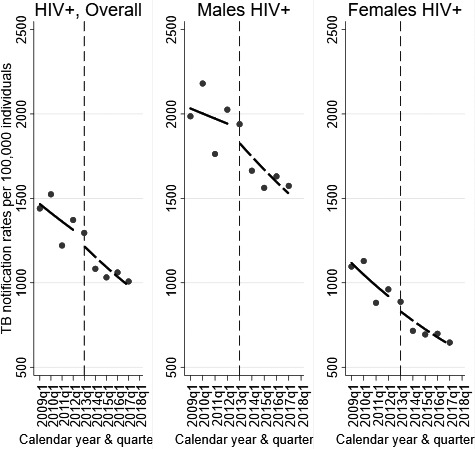
TB notification trends among PLHIV pre‐ and post‐2013 stratified by sex. Dots represent observed rates, solid line present predicted TB notification rates before eMTCT, Dashed lines indicate predicted notification rates after eMTCT from a Poisson regression, Vertical broken line indicate eMTCT implementation (2013).

Poisson regression analysis showed that the differential increase in ART coverage after rollout of eMTCT did not result in faster declines in TB notification rates among HIV positive women. The average decline in NR among HIV‐positive women remained constant at −6.2% (95%CI, −8.1% to −4.3%) before 2013 and −6.6% (95%CI, −8.9% to −4.2%) after 2013 (*p *=* *0.84) (Table 3, Figure 3). Among HIV‐positive men, the average decline in NR increased, although not significantly, from −1.5% (95%CI: −3.5% to +0.6%) before 2013 to −4.4% (−6.7% to −2.0%) after 2013 (*p *=* *0.09) (Table 3, Figure 3).

Among the subset of patients receiving care at the IDI clinic, data from 5742 patients who initiated ART during the study period were analysed. Overall, median baseline CD4+ T‐cell counts increased from 132 (IQR 50 to 204) cells/mL in 2009 to 392 (IQR 131 to 629) cells/mL in 2017 (*p < *0.01). Comparing the periods before and after 2013, median baseline CD4+ T‐cell counts among women increased from 142 (IQR 62 to 209) cells/mL to 240 (IQR 112 to 328) cells/mL (*p < *0.01) before 2013 and from 240 (IQR 112 to 328) cells/mL to 450 (IQR 168 to 704) cells/mL (*p < *0.01) after 2013. Among men, the median baseline CD4+ T‐cell counts increased from 101 (IQR 33 to 193) cells/mL to 216 (IQR 86 to 299) cells/mL (*p < *0.01) before 2013 but only from 216 (IQR 86 to 299) cells/mL to 273 (IQR 79 to 584) cells/mL (*p *=* *0.15) (Figure S1).

**Figure 4:**
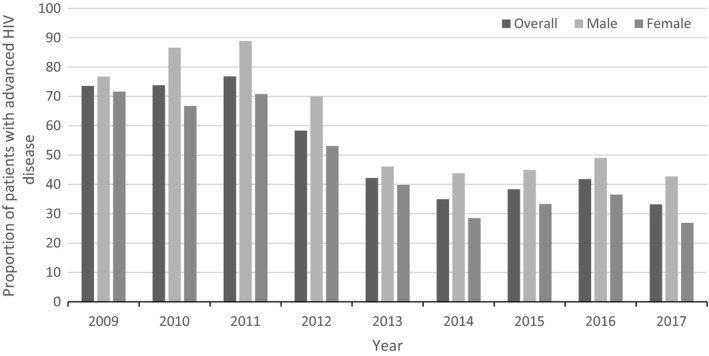
Proportion of patients with advanced HIV disease (CD4 <200) at ART initiation stratified by sex for the period 2009 to 2019.

The proportion of patients initiating ART with advanced HIV disease declined rapidly from 73.6% in 2009 to 33.2% in 2017 (*p < *0.01) Figure 4. Comparing the periods before and after 2013, the proportion of patients initiating ART with advanced HIV disease decreased from 73.6% to 42.2% before 2013 (*p < *0.01) but only decreased from 42.2% to 32.2% after 2013 (*p = *0.05). Stratified by sex, the proportion initiating ART with advanced HIV disease among women decreased from 71.6% to 39.8% (*p < *0.01) before 2013 but only from 39.8% to 26.9% after 2013 (*p = *0.09). Among men, the proportion initiating ART with advanced HIV disease decreased from 76.8% to 46.6% (*p < *0.01) before 2013 but only from 46.6% to 42.7% after 2013 (*p = *0.5) (Figure 4).

## 4 Discussion

We examined the temporal association between ART scale‐up and TB notification rates overall and stratified by HIV status and sex in central Uganda using data reported to the national TB and AIDS control programs. ART coverage among PLHIV substantially increased from 20% to 50% during the study period and was comparable to that within the Eastern and Southern African region (66% [95% CI 52% to 77%] 7 but remained substantially below the 90% target set by UNAIDS in 2014. There were greater increases in ART coverage among women possibly due to interventions targeting increased HIV testing and ART initiation among pregnant mothers, for example, elimination of mother to child transmission of HIV (eMTCT) that have been robustly scaled‐up in Uganda since 2013 20. Lower rates of ART uptake, among men could be a result of poorer health seeking behaviour. Studies from Uganda and other similar settings have shown that men are less likely to; test for HIV, initiate ART following a positive test and achieve virologic suppression while on ART 17, 31, 32. This may be attributed to structural barriers, for example, long working hours that increase difficulty in accessing healthcare, or cultural beliefs about male masculinity that discourage health seeking behaviour 33.

TB NRs were higher among men throughout the study period and did not decrease with increased ART coverage. Higher TB NRs among men have been attributed to a higher burden of TB which could be explained by biological differences 34, 35 and/or differential exposure to various social and occupational risk factors for TB, for example, smoking and alcohol consumption. The population based TB prevalence survey carried out in Uganda in 2015 showed that TB prevalence among men was 686/100,000 population compared to 169/100,000 population among women 36. This male‐to‐female TB prevalence ratio, approx. 4:1, was the second‐highest recorded among prevalence surveys carried out in that period 37 In addition, a meta‐analysis of data from prevalence surveys carried out in Africa, South East Asia and the Mediterranean region from 1994 to 2010 showed that TB prevalence was twice as high among men as women. Higher TB prevalence among men could also be explained by differential access to healthcare services for both TB and HIV. In our analysis, differential access to HIV care services was demonstrated by the lower ART coverage, the lower CD4 cell counts at ART initiation and a higher proportion of male patients initiating ART with advanced HIV disease throughout the study period. Differential access to TB services in Uganda was shown in a study from South‐western Uganda in which men were more likely to have a higher bacterial load at TB diagnosis than women 38.

Mathematical modelling has predicted increasing ART coverage to be temporally associated with a lower incidence of primary and recurrent TB among PLHIV particularly when initiated at higher CD4+ T‐cell counts 13, 14. Among HIV‐positive women, TB NRs decreased steadily throughout the study period with higher rates of decrease seen before 2013 probably due to a combination of increasing ART coverage, higher CD4+ T‐cell counts at ART initiation and a progressively lower proportion of patients initiating ART with advanced HIV disease. However, after 2013, there was no further increase in the rate of decline in TB NRs despite increasing ART coverage and increasingly higher CD4+ T‐cell counts at ART initiation. This result may be attributed to the fact that there were no further decreases in the proportion of women initiating ART with advanced HIV disease in that period. Patients with advanced HIV disease are particularly at risk for several opportunistic infections, including TB.

The annual rates of decline in TB notifications observed in our study (3.5%) are much lower than the 10% annual rate of decline required to reach the End TB targets. This is consistent with another study from South Africa which showed annual rates of decline in TB notifications of 5% following ten years of ART scale‐up in which ART coverage increased from 0% to 63% 39, as well as a recent analysis of trends across sub‐Saharan Africa using UNAIDS and WHO country‐wide estimates 40. These studies show that ART scale‐up alone may be not sufficient to end the TB epidemic in sub‐Saharan Africa. In order to achieve a 90% reduction in TB prevalence by 2030, sub‐Saharan African countries, including Uganda should not only focus on reaching the second 90 through increased ART provision but should place equal emphasis on earlier HIV detection and ART initiation. In addition, other interventions for TB prevention, for example, provision of isoniazid preventive therapy (IPT), which is effective in preventing TB, even in patients with CD4+ T‐cell counts >500 cells/mm 41 and remains sub‐optimally implemented, should be scaled‐up.

This study used notification data from the NTLP. Although quality assurance measures are taken by the NTLP to ensure completeness, missing data remain a limitation of this study. Other limitations include the possibility of reporting bias since patients may travel into the capital (Kampala) in search of better healthcare services from districts besides those included in this study. However, analysis of patient level data for Kampala district carried out by the NTLP showed that more than 85% of TB cases notified by Kampala are from the districts included in this analysis *(personal communication, NTLP Data Manager)*. Finally, the decreases in TB notifications to the NTLP may result from other changes in TB and HIV service delivery other than the availability of ART. However, the introduction of interventions aimed at increasing TB detection, for example, the decrease in number of sputum samples required for diagnosis and the introduction of a more sensitive diagnostic test (Xpert MTB/RIF) during the study period make this unlikely.

## 5 Conclusions

Although ART scale‐up was temporally associated with a decline in tuberculosis notification rates, the achieved rates of decline are below those required to achieve a 90% reduction in tuberculosis incidence by 2030. Additional investments in tuberculosis control should include efforts to promote earlier care seeking and ART initiation among HIV‐positive persons.

## Competing interest

The authors have no competing interest to declare.

## Authors’ contributions

SH and YCM conceived the study. SZM, FRM and JMR collected the data. SZM, JM, YCM and SH analysed and interpreted the data. All authors contributed to the drafting and final review of the manuscript.

## Supporting information


**Table S1.** Numbers and rates of persons notified with TB over time, overall and stratified by HIV status plus ART coverage
**Figure S1.** Median baseline CD4 cell counts at ART initiation stratified by sex for the period 2009 to 2017
