# Supplementary material for: Anti‐retroviral therapy scale‐up and its impact on sex‐stratified tuberculosis notification trends in Uganda
Source: J Int AIDS Soc. 2019 Sep 16;22(9):e25394. doi: 10.1002/jia2.25394 (PMC6747005; doi:10.1002/jia2.25394)
Supplement: Supplementary file 1 — Table S1. Numbers and rates of persons notified with TB over time, overall and stratified by HIV status plus ART coverage Figure S1. Median baseline CD4 cell counts at ART initiation stratified by sex for the period 2009 to 2017 [file JIA2-22-e25394-s001.docx]

**Supplementary Table 1:** N**umbers and rates of persons notified with TB over time, overall and stratified by HIV status plus ART coverage**

| **Year** | **Quarter^a^** | **Overall** | | | | **HIV-negative persons** | | | | **HIV-positive persons** | | | | **ART coverage** | |
| --- | --- | --- | --- | --- | --- | --- | --- | --- | --- | --- | --- | --- | --- | --- | --- |
|  |  | **Males** | | **Females** | | **Males** | | **Females** | | **Males** | | **Females** | | **Males** | **Females** |
|  |  | **No^b^.** | **NR^c^** | **No** | **NR** | **No.** | **NR** | **No.** | **NR** | **No.** | **NR** | **No.** | **NR** | **No. (%)** | **No. (%)** |
| 2009 | Q1 | 1729 | 296 | 1316 | 215 | 900 | 165 | 641 | 116 | 829 | 2182 | 675 | 1120 | 9297 (6) | 17118(8) |
| 2009 | Q2 | 1637 | 278 | 1294 | 210 | 897 | 163 | 630 | 114 | 740 | 1930 | 664 | 1092 | 15495 (10) | 28530(13) |
| 2009 | Q3 | 1768 | 297 | 1257 | 202 | 1055 | 190 | 617 | 111 | 713 | 1843 | 640 | 1043 | 27892(18) | 51354(22) |
| 2009 | Q4 | 1687 | 281 | 1146 | 183 | 909 | 162 | 447 | 80 | 778 | 1993 | 699 | 1129 | 28433(18) | 52533(23) |
| 2010 | Q1 | 1646 | 272 | 1219 | 193 | 791 | 140 | 517 | 92 | 855 | 2091 | 702 | 1082 | 28974(18) | 53712(22) |
| 2010 | Q2 | 1722 | 283 | 1259 | 198 | 836 | 147 | 502 | 88 | 886 | 2148 | 757 | 1089 | 29515(18) | 54891(22) |
| 2010 | Q3 | 1874 | 305 | 1360 | 212 | 884 | 154 | 533 | 93 | 990 | 2379 | 827 | 1179 | 30055(18) | 56071(22) |
| 2010 | Q4 | 1804 | 291 | 1270 | 196 | 922 | 160 | 449 | 78 | 882 | 2101 | 821 | 1161 | 33117(20) | 59604(24) |
| 2011 | Q1 | 1782 | 285 | 1260 | 193 | 1014 | 175 | 619 | 107 | 768 | 1732 | 641 | 905 | 36179(21) | 63137(23) |
| 2011 | Q2 | 1915 | 304 | 1267 | 193 | 995 | 170 | 598 | 102 | 920 | 2057 | 669 | 937 | 39241(22) | 66670(25) |
| 2011 | Q3 | 1802 | 284 | 1170 | 177 | 966 | 164 | 506 | 86 | 836 | 1854 | 664 | 922 | 42303(24) | 70204(26) |
| 2011 | Q4 | 1547 | 242 | 1127 | 169 | 904 | 152 | 572 | 96 | 643 | 1414 | 555 | 764 | 44566(24) | 73751(27) |
| 2012 | Q1 | 2045 | 317 | 1374 | 204 | 1108 | 185 | 576 | 96 | 937 | 2051 | 798 | 1098 | 46829(26) | 77299(28) |
| 2012 | Q2 | 1860 | 286 | 1260 | 186 | 932 | 154 | 570 | 95 | 928 | 2015 | 690 | 942 | 49092(27) | 80745(29) |
| 2012 | Q3 | 2004 | 306 | 1276 | 187 | 1079 | 177 | 646 | 106 | 925 | 1992 | 630 | 853 | 51354(28) | 84394(30) |
| 2012 | Q4 | 1839 | 279 | 1177 | 171 | 883 | 144 | 466 | 76 | 956 | 2042 | 711 | 955 | 53514(29) | 91431(32) |
| 2013 | Q1 | 1975 | 297 | 1335 | 192 | 951 | 154 | 564 | 91 | 1024 | 2158 | 771 | 1028 | 54056(29) | 96023(34) |
| 2013 | Q2 | 1803 | 269 | 1326 | 190 | 894 | 144 | 585 | 94 | 909 | 1901 | 741 | 981 | 54599(29) | 100614(35) |
| 2013 | Q3 | 1894 | 281 | 1293 | 184 | 996 | 159 | 733 | 117 | 898 | 1863 | 560 | 735 | 59993(31) | 114015(40) |
| 2013 | Q4 | 1887 | 279 | 1184 | 168 | 995 | 158 | 566 | 90 | 892 | 1840 | 618 | 809 | 61833(32) | 117756(41) |
| 2014 | Q1 | 1859 | 272 | 1239 | 173 | 1025 | 161 | 602 | 95 | 834 | 1711 | 637 | 826 | 63674(33) | 121497(42) |
| 2014 | Q2 | 1849 | 268 | 1007 | 140 | 1069 | 167 | 512 | 80 | 780 | 1588 | 495 | 637 | 65515(33) | 125238(43) |
| 2014 | Q3 | 1895 | 273 | 1234 | 170 | 1072 | 166 | 714 | 111 | 823 | 1663 | 520 | 664 | 67355(34) | 128979(44) |
| 2014 | Q4 | 1791 | 256 | 1076 | 147 | 945 | 146 | 496 | 76 | 846 | 1693 | 580 | 736 | 70513(35) | 135218(45) |
| 2015 | Q1 | 1765 | 251 | 1026 | 140 | 949 | 145 | 472 | 72 | 816 | 1616 | 554 | 700 | 73671(37) | 141458(47) |
| 2015 | Q2 | 1824 | 261 | 1179 | 159 | 983 | 152 | 576 | 89 | 841 | 1681 | 603 | 756 | 76828(38) | 147697(48) |
| 2015 | Q3 | 1957 | 278 | 1081 | 147 | 1114 | 171 | 497 | 76 | 843 | 1672 | 584 | 740 | 79986(39) | 153936(50) |
| 2015 | Q4 | 1581 | 223 | 899 | 122 | 890 | 135 | 418 | 64 | 691 | 1361 | 481 | 605 | 81572(40) | 156899(51) |
| 2016 | Q1 | 1848 | 255 | 1029 | 136 | 998 | 148 | 466 | 69 | 850 | 1635 | 563 | 691 | 83726(40) | 159862(51) |
| 2016 | Q2 | 1844 | 253 | 1078 | 142 | 977 | 144 | 501 | 74 | 867 | 1655 | 577 | 704 | 84745(40) | 162825(52) |
| 2016 | Q3 | 1890 | 257 | 1145 | 149 | 988 | 145 | 572 | 84 | 902 | 1710 | 573 | 694 | 86331(41) | 165788(53) |
| 2016 | Q4 | 1709 | 231 | 1050 | 136 | 900 | 131 | 465 | 68 | 809 | 1523 | 585 | 704 | 87917(41) | 168751(54) |
| 2017 | Q1 | 1898 | 255 | 1101 | 142 | 1052 | 152 | 518 | 75 | 846 | 1581 | 583 | 696 | 87352(41) | 169471(55) |
| 2017 | Q2 | 1933 | 258 | 1117 | 143 | 1014 | 146 | 522 | 75 | 919 | 1706 | 593 | 706 | 93435(43) | 182638(58) |
| 2017 | Q3 | 1925 | 255 | 1058 | 134 | 1050 | 150 | 553 | 79 | 875 | 1613 | 505 | 595 | 95023(44) | 187674(59) |
| 2017 | Q4 | 1669 | 220 | 985 | 124 | 906 | 129 | 481 | 68 | 763 | 1397 | 504 | 590 | 99571(46) | 192555(60) |

^a^ *calendar quarter* ^b^ *absolute numbers of TB patients notified* ^c^ *TB notification rates per 100,000 population*

**Supplementary Figure 1: Median baseline CD4 cell counts at ART initiation stratified by sex for the period 2009-2017**

**
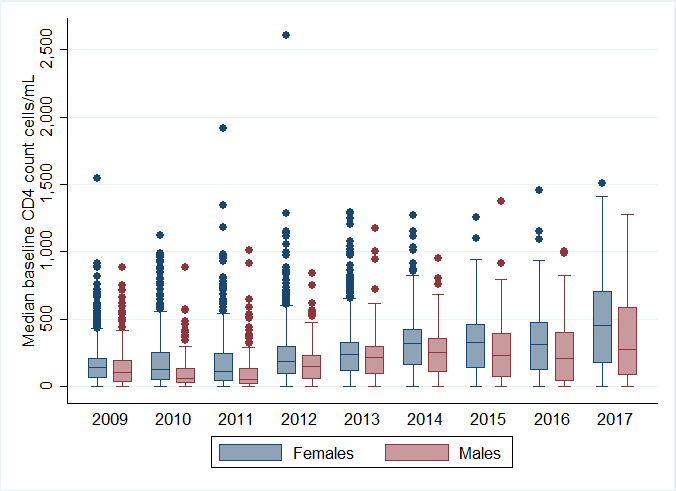
**
